# Involving antimicrobial stewardship programs in COVID-19 response efforts: All hands on deck

**DOI:** 10.1017/ice.2020.69

**Published:** 2020-03-13

**Authors:** Michael P. Stevens, Payal K. Patel, Priya Nori

**Affiliations:** 1 Healthcare Infection Prevention Program, Virginia Commonwealth University Health System, Richmond, Virginia; 2 Infectious Diseases Section, Ann Arbor VA Medical Center, Ann Arbor, Michigan; 3 Division of Infectious Diseases, Department of Medicine, Albert Einstein College of Medicine, Montefiore Medical Center, Bronx, New York

*To the Editor*—To our knowledge, no formal recommendations exist for the inclusion of antimicrobial stewardship programs (ASPs) in disaster planning or emergency response preparedness efforts.^1^ A PubMed search utilizing the search terms “antimicrobial stewardship” AND “disaster planning” was performed on March 4, 2020, and yielded no results. ASPs are now ubiquitous. They often include pharmacists and physicians with advanced infectious diseases training, and they are a valuable part of hospital safety and quality programs. In some hospitals, compartmentalization of stewardship and epidemiology functions have developed over time to meet distinct institutional needs. However, domains should coalesce for purposes of emergency preparedness. The current SARS-CoV-2/COVID-19 outbreak highlights numerous opportunities where ASPs can support emerging pathogen response and planning efforts.

**Fig. 1.**
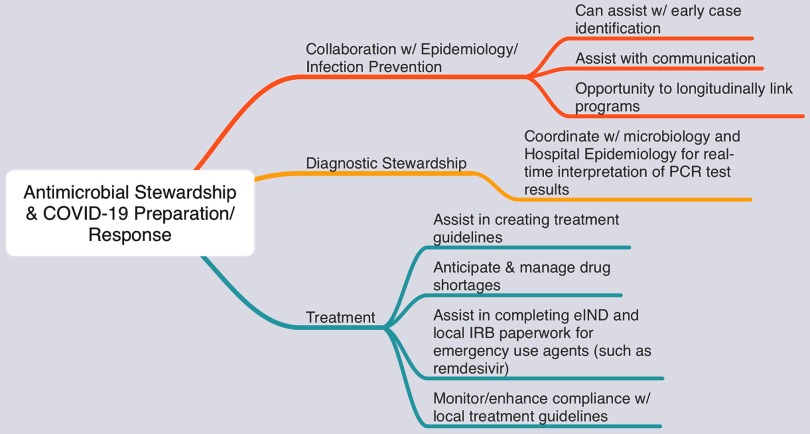
Opportunities for antimicrobial stewardship programs to assist COVID-19 response preparation and planning efforts.

An informal Twitter poll was initiated on March 1, 2020, asking the infectious diseases and antimicrobial stewardship communities whether ASPs at their health systems had been involved in SARS-CoV-2/COVID-19 outbreak response or preparation. This yielded 254 responses: 30% noted direct involvement, 28% indicated indirect involvement, and 39% indicated no involvement in emergency response efforts or planning. Although formalized study is needed, real-time insights from the community provided valuable information. We identified multiple potential areas where ASPs can support emergency response efforts, and these are summarized in Figure 1.

ASPs that are integrated with hospital infection prevention programs have an advantage in response efforts to emerging pathogens in that (1) they are likely to have pre-existing infection prevention skills and experience, (2) they are likely to be involved in response efforts early, and (3) they will have access to and influence with key stakeholders. Because ASPs and infection prevention programs share similar technology infrastructure, data, and metrics, program integration has many advantages.^2^ Response efforts to novel respiratory viruses like SARS-CoV-2/COVID-19 represent an opportunity for programs to formally integrate, to develop cross-coverage capabilities, and to create shared leadership opportunities.

ASPs can support SARS-CoV-2/COVID-19 response efforts in numerous ways within the context of their normal daily activities. A core component of antimicrobial stewardship includes postprescriptive review with feedback to providers.^3^ In this way, an ASP skill set can theoretically assist with early identification of potential cases. This approach may be especially useful in situations in which the definition of a person under investigation is fluid because traditional epidemiologic efforts usually focus on identifying patients at the point of entry into health systems. ASPs often coordinate with microbiology laboratories for real-time interpretation and action involving upper respiratory PCR test results. They can support SARS-CoV-2/COVID-19 evaluation efforts in this fashion as well. Novel respiratory virus outbreaks associated with secondary bacterial pneumonias and acute respiratory distress syndrome (ARDS) provide an opportunity for ASPs to monitor compliance with guideline-concordant therapy; severe COVID-19 cases have been treated with broad-spectrum antibiotics.^4^

Additionally, ASPs can help in the development of local treatment protocols involving repurposed antivirals; they can monitor and manage drug shortages due to supply chain interruptions^5^; and they can assist frontline providers with expanded access investigational new drug applications (eINDs) and local institutional review board procedures for investigational agents.

ASPs are now mandated in the United States and are often multidisciplinary. The Joint Commission accreditation standard for ASPs includes, when available, an infectious diseases physician, pharmacist, infection preventionist, and other practitioners.^6^ ASP physician and pharmacy leaders often have specialized infectious diseases training.^3^ Leveraging these resources for planning and response efforts for emerging pathogens is critical and can strengthen and sustain collaborative relationships.

We recommend that hospital epidemiology programs strongly consider integrating their ASP colleagues into disaster preparedness plans as well as identify a more formal role for stewards in their operations beyond the current COVID-19 outbreak.
